# 

**Published:** 1894-05-26

**Authors:** 


**Notices of Births, Deaths, and Marriages** are inserted in this column at a charge of 2s. 6d. for an announcement not exceeding four lines.

**Notice to Advertisers and Subscribers.**

All Advertisements, Orders for Copies of The Hospital, and Business Communications should be addressed to **The Publisher, NOT TO THE EDITOR,** The Hospital, 428, Strand, W.C.

The Cost of Subscription, post free, is as follows:—Per week, 2½d.; per quarter, 2s. 8d.; per half-year, 5s. 3d.; per annum, 10s. 6d. Foreign:—Per Annum, 12s. 8d.; payable, in all cases, in advance.

### NOTICE TO CORRESPONDENTS.

All MS., Letters, Books for Review, and other matters intended for the Editor should be addressed The Editor, The Lodge, Porchester Square, London, W.

The Editor cannot undertake to return rejected MS., even when accompanied by stamped directed envelope.

**"Burdett's Hospital Annual," 1894.**

Fifth year of publication. 900 pp. Boards, gilt lettering. A most complete guide to British, Colonial, Indian, and American Hospitals, Dispensaries, Nursing and Convalescent Institutions, and Asylums. Price 5s. Published by The Scientific Press (Limited), 428, Strand, W.C.

**NOTICE.—The Index for Vol. XIV. (ending Sept. 30th, 1893) is on sale at the Office of this Journal, price 2½d., post free.**

### HOSPITAL FOR EPILEPSY AND PARALYSIS.

### SCRAPS AND GLEANINGS.

A concert has just been given to the inmates of the Edinburgh Royal Infirmary by Mr. Albert Chevalier.

The degree of D.C.L. was conferred at Oxford on Professor August Weismann, on the occasion of his public lecture in that city.

A balance on the right side of £41 has been carried on to next year's working of the Derbyshire Convalescent Home, an institution deserving of all support.

The Dublin Royal Hospital for Incurables has been benefited by a gift of £21 from Mrs. Fane Vernon towards the building fund of the institution, and a further donation of £21 from Miss Annie Fox.

The first meeting of the Wirral Joint Hospital Board after election was held recently at Birkenhead. There have been 79 cases of an infectious character treated in the hospital during the year. The total expenditure has been £1,574.

The 12th annual meeting of the Sydney Cottage Hospital showed in its report that the financial condition of the institution was scarcely of so gratifying a nature as that for 1892, but a balance, though small, is carried forward to next year. Mr. W. Rogers, of Sydney, has bequeathed a legacy of £500 to the hospital.

### MEDICAL VACANCIES AND APPOINTMENTS.

### APPOINTMENTS.

Aslett Baldwin, L.R.C.P.Lond., M.R.C.S.Eng., appointed House Surgeon to the Middlesex Hospital. J. E. Beggs, B.A., M.B., B.C.Camb., D.P.H., appointed Assistant Medical Officer at the North-Eastern Hospital, Tottenham. Dr. Chalmers, appointed Medical Officer for the Infectious Diseases Hospital at Bannockburn. J. Culross, M.A., M.B., C.M.Glasg., appointed Medical Officer to the Newton Abbot Union. B. Dawson, M.D., B.Sc., M.R.C.P.Lond., appointed Medical Registrar to the London Hospital. Mr. Emerson, appointed Assistant House Surgeon to the Guest Hospital, Dudley. S. W. Haynes, M.B., appointed Honorary Anæsthetist to the Birmingham and Midland Eye Hospital. R. R. Leatham, B.A., M.B., B.Ch., R.U.I., appointed Assistant Resident Medical Officer to the North-West London Hospital. J. F. Sarjeant, M.R.C.S.Eng., L.R.C.P.Lond., appointed Resident Medical Officer to the North-West London Hospital. W. R. Tuckett, M.R.C.S.Eng., appointed Medical Officer to the Charnwood Forest Convalescent Home.

### VACANCIES.

The following vacancies are announced this week, the amount of salary in each case being given after the name of the institution:

### Resident Medical Officers.

Bridgwater Infirmary.—House Surgeon. Salary £70 per annum, with board and residence. Applications to Mr. John Coombs, Honorary Secretary, by June 15th.

Durham County Hospital.—House Surgeon. Salary £100 per annum, with board and lodging. Appointment for two years. Applications to V. K. Cooper, Honorary Secretary, 16, South Bailey, Durham, by June 1st.

Flintshire Dispensary.—Resident House Surgeon. Salary £120 per annum, with furnished house (rent and taxes free), and coal, light, water, and cleaning, or in lieu thereof £20 per annum; knowledge of Welsh desirable. Applications to W. T. Cole, Secretary, Board Room, Bangillt Street, Holywell, by June 5th.

General Hospital, Birmingham.—Assistant House Surgeon. Board, residence, and washing provided. No salary. Applications to the House Governor by May 26th.

Glasgow Maternity Hospital, 146, Buchanan Street, Glasgow.—Indoor and Outdoor Surgeons. Applications to Arthur Forbes, Secretary, by June 9th.

Great Northern Central Hospital, Holloway Road, N.—Junior House Surgeon. No salary; board, apartments, and laundry provided. Applications to the Secretary by May 28th.

Hospital for Sick Children, 18, Royal Arcade, Newcastle-on-Tyne.—Resident Medical Officer; doubly qualified. Salary £60 per annum, with board, lodging, and laundry. Applications to Robert J. Gibson, Secretary, by May 31st.

Jaffray Suburban Branch of the General Hospital, Gravelly Hill, near Birmingham.—Resident Medical and Surgical Officer; doubly qualified. Salary £150 per annum, with board, residence, and washing. Applications to the House Governor, General Hospital, Birmingham, by May 29th.

New Hospital for Women, 144, Euston Road.—Female Resident Medical Officer and Female Clinical Assistant for Out-patient Department. Applications to the Secretary by May 26th.

North Staffordshire Infirmary and Eye Hospital, Hartshill, Stoke-upon-Trent.—Honorary Assistant House Surgeon. Applications to the Secretary by June 5th.

Royal Free Hospital, Gray's Inn Road, W.C.—Two Resident Medical Officers; doubly qualified. No salary; board, lodging, and washing provided. Applications to the Secretary by June 2nd.

### Non-Resident Medical Officers.

Great Northern Central Hospital, Holloway Road, N.—Casualty Officer; must reside in the immediate neighbourhood of the hospital. Honorarium at the rate of 50 guineas per annum. Applications to William T. Grant, Secretary, by May 28th.

Grosvenor Hospital for Women and Children, Vincent Square, Westminster.—Anæsthetist. Applications to the Secretary by June 1st.

Hospital for Epilepsy and Paralysis and other Diseases of the Nervous System, 32, Portland Terrace, Regent's Park, N.W.—Physician to Out-patients. Applications to the Secretary by June 8th.

Royal South London Dispensary, St. George's Cross, S.E.—House Surgeon; non-resident. Salary £60 per annum. Applications to the Committee of Management by May 28th.

University College, Liverpool.—George Holt Chair of Pathology and Derby Chair of Anatomy. Endowment, £375 per annum each, with share of fees. Applications to the Registrar by June 2nd.

